# Differences in office-based personal space perception between British and Korean populations

**DOI:** 10.3389/fpsyg.2023.1043088

**Published:** 2023-03-22

**Authors:** Mike Richardson, Crescent Jicol, Gerald Taulo, Jaehyun Park, Hyun K. Kim, Michael J. Proulx, Alexandra A. de Sousa

**Affiliations:** ^1^CREATE Lab, Department of Psychology, University of Bath, Bath, United Kingdom; ^2^Department of Computer Science, University of Bath, Bath, United Kingdom; ^3^Centre for Health and Cognition, Bath Spa University, Bath, United Kingdom; ^4^Department of Industrial and Management Engineering, Incheon National University, Incheon, Republic of Korea; ^5^School of Information Convergence, Kwangwoon University, Seoul, Republic of Korea; ^6^Department of Psychology, University of Bath, Bath, United Kingdom

**Keywords:** virtual reality, culture, personal space, spatial cognition, workplace density

## Abstract

We sought to understand how the perception of personal space is influenced by different levels of social density, spatial density, and type of window-view in South Korean and United Kingdom workplaces. We employed virtual reality to simulate shared and single occupancy offices. We obtained personal space estimations using a virtual disc around the participant which could be extended and retracted, inside the simulation, to indicate perceived amount of personal space, and compared this measure to questionnaire-based estimations. We found that in both cultures participants experienced greater perceived personal space (1) when in a sparse rather than dense office and (2) having a view of the city outside the office. However, British, but not Korean, participants had significantly higher personal space estimations in single occupancy offices than in shared offices. These results suggest subtle cross-cultural differences in workplace experience, that could only be investigated using virtual reality.

## 1. Introduction

Office workers make up a substantial percentage of the workforce (Church et al., 2011). With economic growth comes more employment opportunities and an increase in the number of office workers; it is natural that the development of new office buildings follow a similar trend. However, as the work spaces are designed, there are several considerations that can have a dramatic effect on the productivity of workers and, the companies which they represent (Paradise et al., 2018). In recent years, there has been a strong preference noted for the use of shared workspaces in an effort to reduce costs and foster a collective attitude for collaboration (vanDuinkerken and MacDonald, 2013; Richardson et al., 2017). Additionally, while the flexibility of working from home initiated by the onset of the COVID-19 pandemic had many positive outcomes (Ipsen et al., 2021), hybrid working models are stillpreferred (Babapour Chafi et al., 2021; Pataki-Bittó and Kapusy, 2021).

Benefits, such as flexible use of space and facilitation of collaboration, support the adoption of shared office space within organizations (vanDuinkerken and MacDonald, 2013); in contrast, others argue that the increase in noise and distraction, and a reduction of autonomy are considerable disadvantages (Kim and de Dear, 2013; Nikolaeva and Dello Russo, 2017). A recent review found that a shared office workspace could have negative effects on the health and wellbeing of workers (Richardson et al., 2017). However, the majority of the research in office space design has been conducted within North America and may not generalize well to European or Asian populations. This is particularly relevant when considering spatial cognition in the workplace, as major global companies are likely to have office spaces situated in all the countries they operate in. It is, therefore, important to gain an understanding of cross-cultural differences regarding satisfaction with the workplace.

Virtual reality (VR) is increasingly becoming a useful tool for a wide range of activities, including design work (Gill and Lange, 2015). Within VR, a building developer can allow individuals to step inside any design while adjusting certain aspects to find the perfect layout. Additionally, a VR simulation can be used anywhere in the world, and will appear identical whether viewed in Europe or Asia; allowing for direct comparison between cultures, without the logistical trouble of moving people or modelled spaces. An architect with the means to quickly, and realistically, simulate a wide range of designs could draw on cognitive research to tailor design specifically around human spatial processing and improve contentedness with built environments (Proulx et al., 2016). VR for design work is not without its drawbacks, for example, people perceive space differently in real and virtual environments (Iachini et al., 2016); meaning direct translation may not be appropriate. However, as there are many factors that interplay personal space perception, such as, gender, age facial expressions, and threatening stimuli (e.g., Coello et al., 2012; Iachini et al., 2016; Ruggiero et al., 2017), VR does offer an ideal testbed for rapidly prototyping the effect of environmental characteristics on spatial cognition.

There is perhaps an argument to be made about whether certain cultures require less personal space in a work environment because their home life is more closely connected. It has been suggested that some Asian countries are well known for a sense of family togetherness and collectivism (Li and Cheng, 2015), and as such live in close proximity, particularly in rural areas (Zimmer and Korinek, 2008). However, for several past generations, the typical western household has reduced the number of family members under one roof, and increased the amount of space per individual. Indeed, as Americans lose personal connections with family and friends, they inversely gain square footage of home space (McKibben, 2007). Perhaps, this preference for space in home life may also spill over into work life.

The personal space an individual feels in the workplace could be influenced by social density (Sinha and Sinha, 1991; Dickinson et al., 2019). For example, a worker in a large single occupancy office could be described as having a low social density (Duval et al., 2002); conversely, a large group of workers in a small office could be described as having a high social density. There is surprisingly little known about differences in work-place personal space preferences cross-culturally, despite the similarities in office design between South Korea and the United Kingdom; a Korean (British) person could easily recognize a British (Korean) office by universal office features like computers on desks, internal walls or cubicles, and desk chair placements.

The presence of a window can have a dramatic effect on psychological health and wellbeing (Ulrich, 1984), and more specifically, the placement of a window within the workplace has shown to improve job satisfaction and happiness (Lottrup et al., 2015). Glass could possibly alter the perceived spaciousness of a room, as the barrier of the glass is visually permeable (Marquardt et al., 2015). Regarding to personal space, a question could be posed as to whether replacing a wall with glass increases a sense of space (for example, to a view of other offices), or perhaps whether a view to outside the building further increases space in comparison to no windows at all. One particular area of interest is whether a view can mediate any negative effects of increased social density, as this could change how architects plan room construction.

The majority of previous work on workplace personal space relies on subjective measures, for example, questionnaires about satisfaction with a particular environment (e.g., Hedge et al., 1989; Duval et al., 2002). Solely using questionnaires to investigate cross-cultural differences should be approached tentatively, as the process of translation may confound results. However, the use of VR allows for realistic manipulation and objective measurement. By using a controller, people within the rendered environment can digitally mark where they believe their personal space to end. We aimed to address some of the gaps in workspace research by comparing a Korean and a British population in VR office environments with two personal space measures, indicating both personal space estimation and personal space satisfaction, in response to different social densities (increasing the number of workers per office). Furthermore, we looked at whether the presence of an outside or an indoor window had any modulating effect.

## 2. Method

### 2.1. Participants

Participants were recruited from both the University of Bath, United Kingdom, and Incheon National University, South Korea. In the British sample 20 individuals took part (mean age = 29.16 ± 8.55 years) and in the Korean sample 24 individuals took part (mean age = 21.7 ± 2.1 years). All participants were required to read through an information sheet that described the purpose, details of the task, and then provide written informed consent prior to commencing the experiment. Sample size was chosen in line with guidelines for VR research (Grantcharov et al., 2004) and a power calculation based on Experiment 1 in Jicol et al. (2023).

### 2.2. Design

Data were collected independently by United Kingdom-based, and South Korea-based researchers at their respective universities. The data were then collated into a 3 × 4 × 2 mixed-design ANOVA, examining the effect of view type (no-view, internal-view, outdoor-view), social density (sole-occupancy office, 8-person office, 16-person office, 32-person office), and native culture (Korean, British) on personal space estimation and personal space satisfaction. Social density and view type were measured within-subjects, and then subsequently tested between each population with native culture as the comparison.

Within a VR environment, questions were asked, so as to not interrupt any presence in the reality, and answered using an Oculus remote, to determine personal space estimation and personal space satisfaction. Personal space estimation was determined by presenting to the participant a red virtual circle around them (initially set at 0.5 m in diameter), that they could adjust with the remote to a circumference that they felt encompassed all the space that belonged to them. Personal space satisfaction was determined from three questions, the values of which were averaged to generate one value: ‘I am satisfied with the amount of space I have for myself’, ‘I would not require more personal space than I currently have’, ‘I feel like my co-workers are not invading my personal space’. These questions have been used in previous work (Sundstrom et al., 1980; Duval et al., 2002). Responses were on a sliding scale from strongly agree to strongly disagree, scored out of 100 (i.e., moving the slider to the middle would give a score of 50).

The protocol was used to test the effect of social density and view type, and presented the hypotheses: that a single occupancy office would offer greater perceived personal space than a shared one, and that perceived personal space will be reduced in response to an increased social density (H_1_); and, that an outdoor view would provide the impression of more personal space than an indoor view, and an indoor view would provide more than no view (H_2_). Finally, we hypothesized that the British participants would prefer more personal space than the Korean participants (H_3_).

### 2.3. Materials

#### 2.3.1. Apparatus

The VR office environments were created in Unity (2017, version 1.3), a game design and VR engine. The avatars were created in Adobe Fuse CC (2018, version 1.3), and the Unity coding was written in C# programming language. The environments were presented to the participants *via* an Oculus Rift head-mounted display (HMD); with a maximum refresh rate of 90 Hz and OLED panels with a pixel resolution of 1,080 × 1,200 for each eye. The HMD was powered by an Alienware Area 51 desktop computer, operating on Windows 10, and with an Intel Core i7, 3.4 GHz processor, 16 Gb. of RAM, and a Nvidia 1,080 Ti graphics card with 11 Gb. of GDDR5 memory.

#### 2.3.2. Virtual reality office design

**Figure 1:**
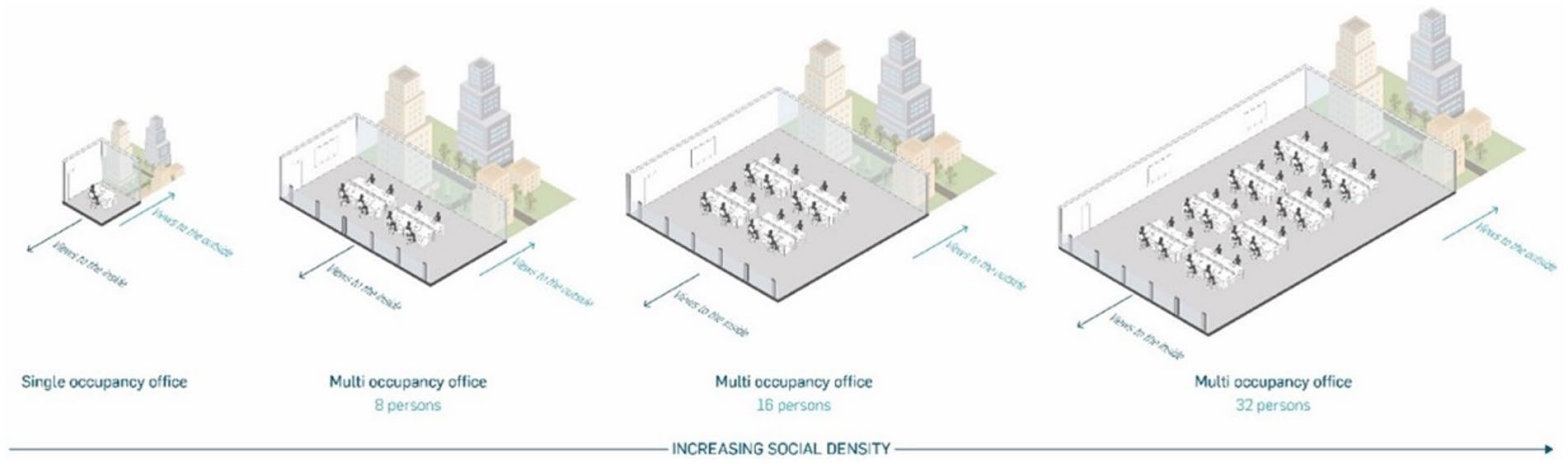
A demonstration of increasing social density with a constant spatial density (the personal space around each person remains the same), for a single occupancy, and an 8-, 16-, and 32-person shared office. Reproduced with permission from Jicol et al. (2023).

When manipulating the social density, the number of occupants in a room was increased, while maintaining a constant amount of space around each person. The offices were separated into four modulations of social density (also in Figure 1).

**Figure 2:**
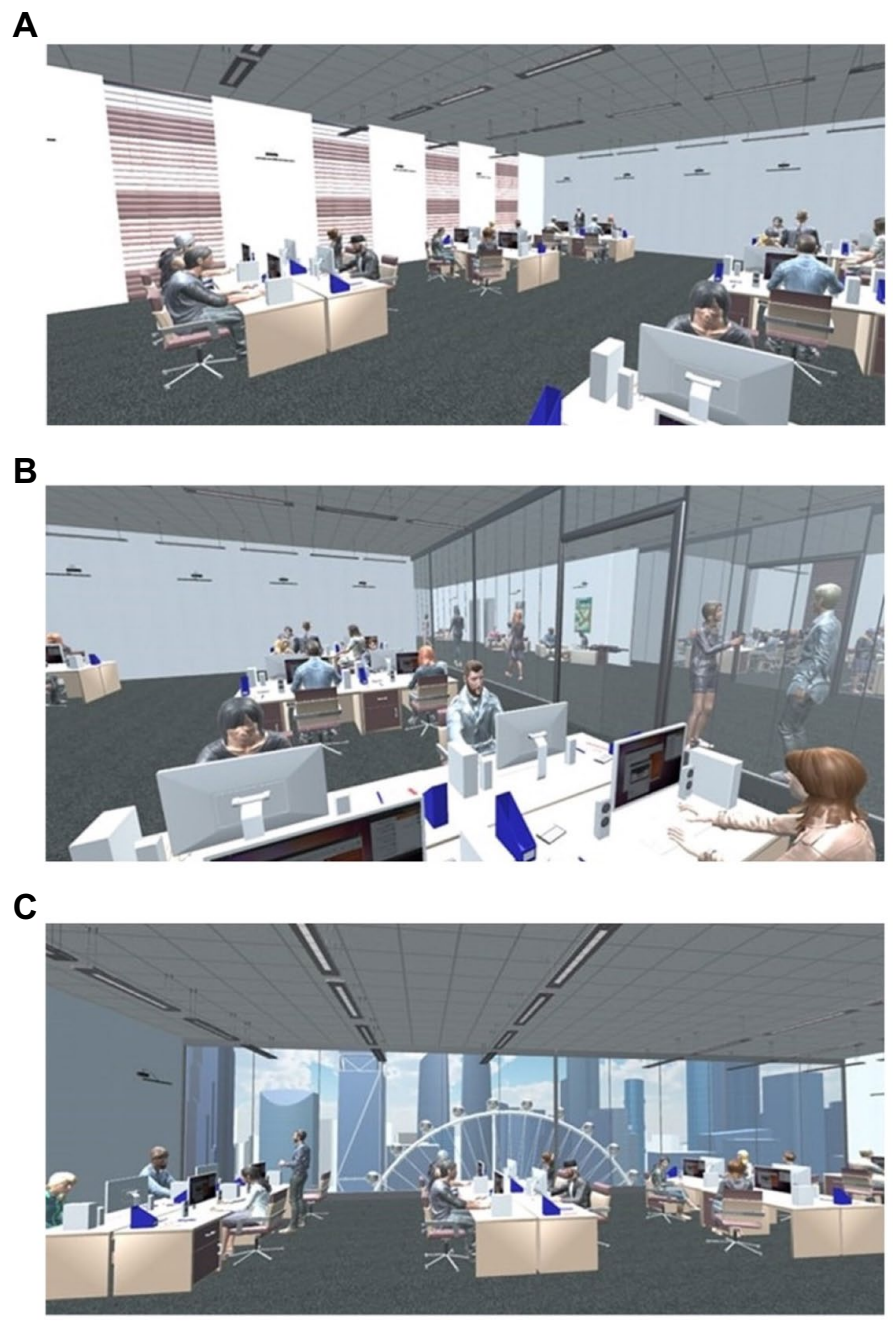
The different office view types from the perspective of the participant. **(A)** = no view office; **(B)** = internal view office; **(C)** = outdoor view office. Reproduced with permission from Jicol et al. (2023).

While the virtual office spaces were identical in both the Korean and United Kingdom populations, the appearance and actions of the avatars were different to appear more naturalistic to both populations: the Korean avatars were modelled to have a Korean appearance, they all wore suits, and, within the office environment, they all sat at their computers typing while looking at the screen. For the United Kingdom population, the avatars were dressed more casually, and their actions varied from typing at a keyboard, to chatting around a watercooler. Participants embodied no avatar (if they were to look down, they would see no body), as this could have evoked confounding factors such as body type or self-reference (Gonzalez-Franco et al., 2019; Figure 2).

#### 2.3.3. Procedure

The procedure follows that of Jicol et al. (2023) which will be described here in brief. All participants provided informed consent, followed by the pre-experiment questionnaire. The experimenter then gave a brief explanation of how the VR headset and remote worked. The online platform Qualtrics was used to host a pre-experimental questionnaire to collect background information. Twelve office spaces were presented randomly for 30 s per environment; the participant was free to move their head to examine each aspect of the office, and they had no virtual body and viewing took place from a stationary position. After the 30 s viewing time was completed, the office environment was replaced by an infinite horizon, as to not provide any distracting spatial cues, which was shown for approximately 10 s. After viewing each office environment, to acquire personal space estimations, the participants were asked to look down to see a red disk that surrounded them. Using the remote, the participants could adjust the disk to the size that they felt encompassed all the space that they ‘owned’. Then, to determine ‘personal space satisfaction’, questions were also posed and participants responded with the sliding scale. Trials were presented in a randomized order, but counterbalancing was not used to keep the number of participants required to lower level. After the study was completed, participants were debriefed. In total, the experiment lasted approximately 30 min.

## 3. Results

To maintain brevity, and avoid the publication of same data multiple times, the results reported in this article are limited to the Korean participant’s only (see Table 1 for mean estimated personal space, and Table 2 for mean questionnaire responses). We also report a cross-paper analysis between the data presented here and the results of experiment two in Jicol et al. (2023). For the United Kingdom sample’s data, refer to the accompanying publication of Jicol et al. (2023).

**Table 1:** Mean and standard deviations of estimated personal space (measured in m^2^) within the virtual environment across view type and social density for the Korean participants.

Social density	No view	Indoor view	Outdoor view
Sole office	1.94 ± 1.42	1.95 ± 0.99	2.00 ± 1.51
8 Person office	1.73 ± 0.80	1.69 ± 0.70	1.71 ± 0.77
16 Person office	1.59 ± 0.53	1.83 ± 0.77	1.64 ± 0.66
32 Person office	1.70 ± 0.60	1.72 ± 0.75	1.62 ± 0.70

**Table 2:** Mean and standard deviations of questionnaire responses concerning personal space satisfaction (scored 0–100) across view type and social density for the Korean participants.

Social density	No view	Indoor view	Outdoor view
Sole office	71.93 ± 16.21	65.04 ± 16.71	77.79 ± 11.93
8 Person office	46.61 ± 19.95	46.13 ± 20.93	48.38 ± 14.30
16 Person office	51.97 ± 19.35	50.18 ± 20.23	52.71 ± 18.08
32 Person office	39.97 ± 21.56	40.51 ± 18.67	47.18 ± 17.95

### 3.1. Korean personal space results

#### 3.1.1. Personal space estimation, social density, and view type

Mauchly’s test for office size × view type indicated the assumption of sphericity had been violated [*χ*^2^ (20) = 95.496, *p* < 0.001, *ε* = 0.446]. The degrees of freedom were corrected using Greenhouse–Geisser estimates of sphericity. For personal space estimation, the repeated measures ANOVA showed there was no significant interaction effect between social density and view type [*F*(2.68, 61.61] = 0.887, *p* = 0.443, *ɳ*_p_^2^ = 0.037).

#### 3.1.2. Personal space estimation and social density

Mauchly’s test for social density indicated that the assumption of sphericity had been violated (*χ*^2^ (5) = 52.852, *p* < 0.001, *ε* = 0.446). The degrees of freedom were corrected using Greenhouse–Geisser estimates of sphericity. Social density did not have a statistically significant main effect on personal space estimation [*F*(1.34, 30.79) = 2.931, *p* = 0.086, *ɳ*_p_^2^ = 0.113].

#### 3.1.3. Personal space satisfaction, social density, view type

Mauchly’s test for social density × view type indicated that the assumption of sphericity had been violated (*χ*^2^ (20) = 31.960, *p* = 0.0.046, *ε* = 0.874). The degrees of freedom were corrected using Huynh-Feldt estimates of sphericity due to a high epsilon value. For personal space satisfaction, there was no significant interaction effect between social density and view type [*F*(5.24, 120.59) = 1.461, *p* = 0.205, *ɳ*_p_^2^ = 0.060].

#### 3.1.4. Personal space satisfaction and social density

Mauchly’s test for social density indicated that the assumption of sphericity had not been violated [*χ*^2^ (5) = 10.385, *p* = 0.065]. Social density had a significant main effect on personal space satisfaction [*F*(3, 69) = 25.649, *p* < 0.001, *ɳ*_p_^2^ = 0.527]. Post-hoc Bonferroni-corrected comparisons demonstrated a significant mean difference for personal space satisfaction between Sole Occupancy and 8-Person Office of 24.55 (95% CI = 15.44, 33.66, *p* < 0.001), between Sole Occupancy and 16-Person Office of 19.97 (95% CI = 8.69, 31.25, *p* < 0.001), between Sole Occupancy and 32-Person Office of 29.03 (95% CI = 17.08, 40.99, *p* < 0.001), and between 16-Person Office and 32-Person Office of 9.07 (95% CI = 1.70, 16.43, *p* = 0.010). No significant difference was found between 8-Person Office and 16-Person Office, and between 8-Person Office and 32-Person Office.

#### 3.1.5. Personal space satisfaction and view type

Mauchly’s test for view type indicated that the assumption of sphericity had not been violated [*χ*^2^ (2) = 2.549, *p* = 0.280]. View type had a significant main effect on personal space satisfaction [*F*(2, 46) = 3.381, *p* = 0.043, *ɳ*_p_^2^ = 0.128]. Post-hoc Bonferroni-corrected comparisons demonstrated a significant mean difference for personal space satisfaction between City View and Internal View of 6.05 (95% CI = 0.97, 11.13, *p* = 0.016). No significant difference was found between No View and Internal View, and between No View and City View.

#### 3.1.6. Summary

For personal space estimation, we found no effect of social density and view type. However, personal space satisfaction was greatest for the single occupancy office; it was perceived as providing more personal space than any of the other three offices. The other three offices were no different amongst themselves except between the 16-Person Office and the 32-Person Office. We found that City View offices scored higher than Internal View offices and that there was no significant difference amongst others.

### 3.2. Effect of culture: Comparing Korean and British space results

#### 3.2.1. Personal space estimation

Levene’s test confirmed that the assumption of the homogeneity of variances had not been violated. The mixed ANOVA demonstrated that culture did not have a statistically significant main effect on personal space estimation [*F*(1, 42) = 2.188, *p* = 0.147, *ɳ*_p_^2^ = 0.050]. There was a significant interaction effect between culture and social density [*F*(1.47, 61.88) = 3.847, *p* = 0.039, *ɳ*_p_^2^ = 0.084]. Note that for the personal space estimation the single occupancy office was the highest for the United Kingdom, but with no difference in all levels of shared office, and there was no difference amongst any of the different social densities in Korea.

#### 3.2.2. Personal space satisfaction

Levene’s test indicated that variances were homogeneous for all levels of repeated measures variables except the eight-Person Office of City View. Because it was the only one of the 12 combinations of levels that was significant, this may not affect the homogeneity of variances overall. Culture had a statistically significant main effect on personal space satisfaction [*F*(1, 42) = 5.147, *p* = 0.028, *ɳ*_p_^2^ = 0.109]. Note that average scores of personal space satisfaction in the United Kingdom and South Korea were 61.1 and 53.2, respectively. There were no significant interaction effects.

#### 3.2.3. Summary

For personal space estimation, culture did not have a significant main effect. But we found a significant effect of culture and social density: Single occupancy offices were estimated to provide significantly more personal space in the United Kingdom, but there was no statistical difference in Korea. However, for personal space satisfaction, culture had a significant main effect in that the British population preferred more space, but there was no difference amongst any interaction effects. Also, for personal space satisfaction in the Korean population, single occupancy offices were preferred.

## 4. Discussion

We found few differences and a number of similarities between the Korean and the British populations. Personal space estimations were significantly higher for single occupancy offices in the British, but not in Koreans. British participants also had higher personal space satisfaction with a lower social density than Korean participants (H_3_ partially accepted). However, while a difference exists between the cultures for personal space satisfaction, no differences were observed in the personal space estimates for different shared office environments—except the single occupancy environment (H_1_ partially accepted). This difference is rather interesting and suggests some dissonance between self-estimated personal space (as indicated by expanding the virtual circle to encapsulate ‘owned’ space) and reported satisfaction with the space (as determine from the questions). The results indicate that a more abstract measure of “personal space satisfaction” may not represent the more concrete measure of “personal space estimation,” but instead could represent self-report bias. For instance, the question ‘I am satisfied with the amount of space for myself’, may be biased by preconceptions for more space equating to more status or power (Konar et al., 1982), rather than referring to how much physical space that person is genuinely happy with. Conversely, the interaction between working environment and socioeconomic status has shown to impact health in both Korean and United Kingdom samples (Macintyre and Hunt, 1997; Lee and Lee, 2019).

Our results are congruent with previous studies that found a difference in attitudes toward work in an office environment between Asian and Western cultures (Bae and Chung, 1997; AMA, 2019). We show that there is benefit in comparing findings outside of Western academic institutions. The lack of observable difference within both personal space estimation and personal space satisfaction with the Korean sample for social density support the notion that Asian cultures experience more ‘togetherness’ (Li and Cheng, 2015), and as such, express less concern when sharing space.

Within the Korean sample, the view type had little effect on the perception of personal space, with no significant result in personal space estimation; within personal space satisfaction, the only observed difference was between a city and internal view. However, as there was no effect between social density and view type, we suggest that while an exterior view is preferred, it does not successfully mediate any negative effects of overcrowding (H_2_ partially accepted). Previous work suggests a strong preference for natural views in comparison to urban ones (Aries et al., 2015), and has advantages for stress relief and for the recovery of physical health (Ulrich, 1984), future research could highlight cross-cultural differences in nature view preference.

One potential limitation of the study was the 30 s viewing time for each office. In pilot testing, 30 s appeared to be enough time to get a good impression of the space, however, it may not have been enough time to achieve a sense of being there (e.g., Zhang et al., 2018). Due to the repeated measures design, it was also impractical to extend the trial length. Additionally, to keep each trial to reasonable length and to reduce the amount of rendering required for each environment, participants viewed from a station position (not moving around the office). Previous work suggests individuals underestimate egocentric distances from a stationary position in VR (Jones et al., 2009). Egocentric spatial judgements are also affected by embodying an avatar (Gonzalez-Franco et al., 2019). We did not include an avatar for the user, to reduce confounds such as mis-matched body types, but this should be considered for future work.

## 5. Conclusion

The present study shows that personal space satisfaction measures previously used may not actually be indicative of true perceptions. However, using VR may also not truly represent individuals in their actual workspaces, although based on past work in other domains, it would seem to be reliable (Peeters, 2019). Regardless, future research would benefit from comparing VR to actual workspaces, to test the validity of using VR to emulate office workspaces. With that said, a large body of work has found that VR can successfully emulate a wide range of environments and paradigms (Kuliga et al., 2015), so while some need for validation is useful, we are relatively confident in the results in relation to actual office workspaces. This report offers one of the first examples of using VR to examine work environments cross-culturally. However, we speculate that the method will become well used due to the benefits of identical space presentation in a format that can be uploaded and downloaded to anyone with a headset, eliminating the need for expensive and time-costly travel or modelling.

## Data availability statement

The raw data supporting the conclusions of this article will be made available by the authors, without undue reservation.

## Ethics statement

The studies involving human participants were reviewed and approved by Department of Psychology Ethics Committee, University of Bath. The patients/participants provided their written informed consent to participate in this study.

## Author contributions

MR conducted the write-up of the manuscript. CJ developed the experimental procedure and conducted data collection. GT contributed to data analysis. JP and HK conducted the Korean aspect of the study. MP and AS advised, supported, and co-developed the experimental protocol and edited the manuscript. All authors contributed to the article and approved the submitted version.

## Funding

MP is supported in part by funding for CAMERA 2.0, the UKRI Center for the Analysis of Motion, Entertainment Research and Applications (EP/T014865/1).

## Conflict of interest

The authors declare that the research was conducted in the absence of any commercial or financial relationships that could be construed as a potential conflict of interest.

## Publisher’s note

All claims expressed in this article are solely those of the authors and do not necessarily represent those of their affiliated organizations, or those of the publisher, the editors and the reviewers. Any product that may be evaluated in this article, or claim that may be made by its manufacturer, is not guaranteed or endorsed by the publisher.

## References

[ref1] AMA. (2019). Survey shows UK job satisfaction and commitment on the decline | AMA. Available at: https://www.amanet.org/articles/survey-shows-uk-job-satisfaction-and-commitment-on-the-decline/.

[ref2] Aries M. B. C. Aarts M. P. J. Van Hoof J. (2015). “Daylight and health: a review of the evidence and consequences for the built environment,” in Lighting Research and Technology. SAGE Publications Ltd. (Vol. 47, 6–27).

[ref3] Babapour Chafi M. Hultberg A. Bozic Yams N. (2021). Post-pandemic office work: perceived challenges and opportunities for a sustainable work environment. Sustain. For. 14:294. doi: 10.3390/su14010294

[ref4] Bae K. Chung C. (1997). Cultural values and work attitudes of Korean industrial workers in comparison with those of the United States and Japan. Work. Occup. 24, 80–96. doi: 10.1177/0730888497024001006

[ref5] Church T. S. Thomas D. M. Tudor-Locke C. Katzmarzyk P. T. Earnest C. P. Rodarte R. Q.. (2011). Trends over 5 decades in U.S. occupation-related physical activity and their associations with obesity. PLoS One 6:e19657. doi: 10.1371/journal.pone.0019657, PMID: 21647427 PMC3102055

[ref6] Coello Y. Bourgeois J. Iachini T. (2012). Embodied perception of reachable space: how do we manage threatening objects? Cogn. Process. 13, 131–135. doi: 10.1007/s10339-012-0470-z, PMID: 22806660

[ref7] Dickinson P. Gerling K. Hicks K. Murray J. Shearer J. Greenwood J. (2019). Virtual reality crowd simulation: effects of agent density on user experience and behaviour. Virtual Reality 23, 19–32. doi: 10.1007/s10055-018-0365-0

[ref8] Duval C. Charles K. Veitch J. A. (2002). Open-Plan Office Density and Environmental Satisfaction Lighting View Project Lighting Quality View Project. National Research Council of Canada: Institute for Research in Construction.

[ref9] Gill L. Lange E. (2015). Getting virtual 3D landscapes out of the lab. Comput. Environ. Urban. Syst. 54, 356–362. doi: 10.1016/j.compenvurbsys.2015.09.012

[ref10] Gonzalez-Franco M. Abtahi P. Steed A.. Individual differences in embodied distance estimation in virtual reality. In: *2019 IEEE Conference on Virtual Reality and 3D User Interfaces (VR)*. IEEE. (2019) p. 941–943.

[ref11] Grantcharov T. P. Kristiansen V. B. Bendix J. Bardram L. Rosenberg J. Funch-Jensen P. (2004). Randomized clinical trial of virtual reality simulation for laparoscopic skills training. BJS (Br. J. Surgery) 91, 146–150. doi: 10.1002/bjs.4407 14760660

[ref12] Hedge A. Burge P. S. Robertson A. S. Wilson S. Harris-Bass J. (1989). Work-related illness in offices: a proposed model of the “sick building syndrome.”. Environ. Int. 15, 143–158. doi: 10.1016/0160-4120(89)90020-2

[ref13] Iachini T. Coello Y. Frassinetti F. Senese V. P. Galante F. Ruggiero G. (2016). Peripersonal and interpersonal space in virtual and real environments: effects of gender and age. J. Environ. Psychol. 45, 154–164. doi: 10.1016/j.jenvp.2016.01.004

[ref14] Ipsen C. van Veldhoven M. Kirchner K. Hansen J. P. (2021). Six key advantages and disadvantages of working from home in Europe during COVID-19. Int. J. Environ. Res. Public Health 18:1826. doi: 10.3390/ijerph18041826, PMID: 33668505 PMC7917590

[ref15] Jicol C. Taulo G. Goldie C. Esenkaya T. Hynes R. Paradise C. Proulx M. de Sousa A. A. (2023). Exploring the effects of environmental cues on perceived personal space in the virtual workplace. Front. Com. Sci. 5. doi: 10.3389/fcomp.2023.1066881

[ref16] Jones J. A. Swan J. E. Singh G. Franck J. Ellis S. R.. The effects of continued exposure to medium field augmented and virtual reality on the perception of egocentric depth. In: *Proceedings of the 6th Symposium on Applied Perception in Graphics and Visualization*. (2009). p. 138–138.

[ref17] Kim J. de Dear R. (2013). Workspace satisfaction: the privacy-communication trade-off inopen-plan offices. J. Environ. Psychol. 36, 18–26. doi: 10.1016/j.jenvp.2013.06.007

[ref18] Konar E. Sundstrom E. Brady C. Mandel D. Rice R. W. (1982). Status demarcation in the office. Environ. Behav. 14, 561–580. doi: 10.1177/0013916582145004

[ref19] Kuliga S. F. Thrash T. Dalton R. C. Hölscher C. (2015). Virtual reality as an empirical research tool—exploring user experience in a real building and a corresponding virtual model. Comput. Environ. Urban. Syst. 54, 363–375. doi: 10.1016/j.compenvurbsys.2015.09.006

[ref20] Lee C. Y. Lee Y. H. (2019). Measurement of socioeconomic position in research on cardiovascular health disparities in Korea: a systematic review. J. Prev. Med. Public Health 52, 281–291. doi: 10.3961/jpmph.19.094, PMID: 31588697 PMC6780291

[ref21] Li T. Cheng S. T. (2015). “Family, friends, and subjective well-being: a comparison between the west and asia” in Friendship and Happiness: Across the Life-Span and Cultures (Netherlands: Springer), 235–247.

[ref22] Lottrup L. Stigsdotter U. K. Meilby H. Claudi A. G. (2015). The workplace window view: a determinant of office workers’ work ability and job satisfaction. Landsc. Res. 40, 57–75. doi: 10.1080/01426397.2013.829806

[ref23] Macintyre S. Hunt K. (1997). Socio-economic position, gender and health: how do they interact? J. Health Psychol. 2, 315–334. doi: 10.1177/135910539700200304 22013025

[ref24] Marquardt G. Cross E. S. de Sousa A. A. Edelstein E. Farnè A. Leszczynski M.. (2015). There or not there? A multidisciplinary review and research agenda on the impact of transparent barriers on human perception, action, and social behavior. Front. Psychol. 6:01381. doi: 10.3389/fpsyg.2015.01381, PMID: 26441756 PMC4569749

[ref25] McKibben B. (2007). Deep Economy: The Wealth of Communities and the Durable Future Macmillan.

[ref26] Nikolaeva R. Russo S. Dello. (2017).“Office design and dignity at work in the knowledge economy,” in Dignity and the Organization. eds. Kostera M. Pirson M. (Macmillan UK: Palgrave), 197–220.

[ref27] Paradise C. Hynes R. Proulx M. J. de Sousa A. A. Jicol C. Esenkaya T. (2018). The psychology of workplace design. Conscious Cities J. 5

[ref28] Pataki-Bittó F. Kapusy K. (2021). Work environment transformation in the post COVID-19 based on work values of the future workforce. J. Corporate Real Estate 23, 151–169. doi: 10.1108/JCRE-08-2020-0031

[ref29] Peeters D. (2019). “Virtual reality: a game-changing method for the language sciences” in Psychonomic Bulletin and Review, vol. 26 (Springer New York LLC), 894–900. 30734158 10.3758/s13423-019-01571-3 PMC6557875

[ref30] Proulx M. J. Todorov O. S. Aiken A. T. de Sousa A. A. (2016). Where am I? Who am I? The relation between spatial cognition, social cognition and individual differences in the built environment. Front. Psychol. 7:00064. doi: 10.3389/fpsyg.2016.00064, PMID: 26903893 PMC4749931

[ref31] Richardson J. C. Maeda Y. Lv J. Caskurlu S. (2017). Social presence in relation to students’ satisfaction and learning in the online environment: a meta-analysis. Comput. Hum. Behav. 71, 402–417. doi: 10.1016/j.chb.2017.02.001

[ref32] Ruggiero G. Frassinetti F. Coello Y. Rapuano M. Di Cola A. S. Iachini T. (2017). The effect of facial expressions on peripersonal and interpersonal spaces. Psychol. Res. 81, 1232–1240. doi: 10.1007/s00426-016-0806-x, PMID: 27785567

[ref33] Sinha S. P. Sinha S. P. (1991). Personal space and density as factors in task performance and feeling of crowding. J. Soc. Psychol. 131, 831–837. doi: 10.1080/00224545.1991.9924670, PMID: 1816468

[ref34] Sundstrom E. Burt R. E. Kamp D. (1980). Privacy at work: architectural correlates of job satisfaction and job performance. Acad. Manag. J. 23, 101–117. doi: 10.5465/255498

[ref35] Ulrich R. S. (1984). View through a window may influence recovery from surgery. Science 224, 420–421. doi: 10.1126/science.6143402, PMID: 6143402

[ref36] vanDuinkerken W. MacDonald K. I. (2013). “Challenges of redesigning staff work space” in Workplace Culture in Academic Libraries: The Early 21st Century. eds. Blessinger K. Hrycaj P. (Elsevier Inc), 147–162.

[ref37] Zhang C. Hoel A. S. Perkis A. Zadtootaghaj S.. How long is long enough to induce immersion. In: *2018 Tenth International Conference on Quality of Multimedia Experience (QoMEX). IEEE*. (2018).p. 1–6.

[ref38] Zimmer Z. Korinek K. (2008). Does family size predict whether an older adult lives with or proximate to an adult child in the Asia-Pacific region? Asian Popul. Stud. 4, 135–159. doi: 10.1080/17441730802246861

